# Melanoma Cell Adhesion and Migration Is Modulated by the Uronyl 2-O Sulfotransferase

**DOI:** 10.1371/journal.pone.0170054

**Published:** 2017-01-20

**Authors:** Katerina Nikolovska, Dorothe Spillmann, Jörg Haier, Andrea Ladányi, Christian Stock, Daniela G. Seidler

**Affiliations:** 1 Institute of Physiological Chemistry and Pathobiochemistry, University of Münster, Münster, Germany; 2 Centre for Internal Medicine, Department of Gastroenterology, Hepatology and Endocrinology, Hannover Medical School, Hannover, Germany; 3 Department of Medical Biochemistry and Microbiology, Biomedical Center, Uppsala University, Uppsala, Sweden; 4 Comprehensive Cancer Center Münster, University Hospital Münster, Münster, Germany; 5 Department of Surgical and Molecular Pathology, National Institute of Oncology, Budapest, Hungary; University of Patras, GREECE

## Abstract

Although the vast majority of melanomas are characterized by a high metastatic potential, if detected early, melanoma can have a good prognostic outcome. However, once metastasised, the prognosis is bleak. We showed previously that uronyl-2-O sulfotransferase (Ust) and 2-O sulfation of chondroitin/dermatan sulfate (CS/DS) are involved in cell migration. To demonstrate an impact of 2-O sulfation in metastasis we knocked-down *Ust* in mouse melanoma cells. This significantly reduced the amount of Ust protein and enzyme activity. Furthermore, *in vitro* cell motility and adhesion were significantly reduced correlating with the decrease of cellular Ust protein. Single cell migration of B16V^shUst(16)^ cells showed a decreased cell movement phenotype. The adhesion of B16V cells to fibronectin depended on α5β1 but not αvβ3 integrin. Inhibition of glycosaminoglycan sulfation or blocking fibroblast growth factor receptor (FgfR) reduced α5 integrin in B16V cell lines. Interestingly, FgfR1 expression and activation was reduced in *Ust* knock-down cells. *In vivo*, pulmonary metastasis of B16V^shUst^ cells was prevented due to a reduction of α5 integrin. As a proof of concept *UST* knock-down in human melanoma cells also showed a reduction in *ITGa*5 and adhesion. This is the first study showing that Ust, and consequently 2-O sulfation of the low affinity receptor for FgfR CS/DS, reduces *Itga*5 and leads to an impaired adhesion and migration of melanoma cells.

## Introduction

A critical event in tumorigenesis of melanoma is the conversion from a primary tumor into an aggressive, metastasizing tumor. Tumor metastasis is a complex process involving its stroma, cell migration and invasion. Cell surface glycans, especially proteoglycans are involved in different stages of metastasis [1, 2]. Proteoglycans are proteins covalently modified by a linear glycosaminoglycan (GAG) chain composed of repeating disaccharide units of an amino sugar and uronic acid [3]. Physiologically, GAGs are involved in multiple cellular functions, such as cell–matrix, cell–cell and ligand–receptor interactions. GAGs such as heparin/heparan sulfate (HS) or chondroitin/dermatan sulfate (CS/DS) can act as low affinity receptor for the biological activity of fibroblast growth factors (FGFs) [1, 4, 5] suggesting that CS/DS might have important regulatory functions [6–8]. CS/DS are galactosaminoglycans composed of N-acetylgalactosamine (GalNAc) and either d-glucuronic acid (d-GlcUA) or l-iduronic acid (l-IdoUA). The inversion of d-GlcUA to l-IdoUA occurs on the polymer level by the chondroitin-glucuronate C5-epimerase (EC 5.1.3.19) (DS-epimerase) [7], first described as SART2, a protein of unknown function over-expressed in cancer cells [9]. The microheterogeneity of CS/DS depends on the presence of (-4GlcUAβ1-3GalNAcβ1-) and (-4IdoUAα1-3GalNAcβ1-) which can be differentially sulfated at C4, C6 (GalNAc) and/or C2 (d-GlcUA/l-IdoUA) by specific sulfotransferases. The minor modification at C2 is introduced by uronyl 2-O sulfotransferase (UST (no EC number) which transfers a sulfate group from 3’-phosphadenosine-5-phosphosulfate. UST is encoded by only one gene (*UST*) [10]. There is evidence that GAG structures are altered during metastasis of melanoma cells due to up-regulation of CS/DS-proteoglycans [2, 11, 12]. Notably, melanoma derived GAGs display a shift from HS and DS to CS in which CS contains high amounts of GlcUA-GalNAc(6S) (ΔdiCS-6S) and ΔdiCS-nS units [13]. Enzymatic digestion of cell surface CS/DS reduces proliferation and invasion of cancer cells [14]. The understanding of the mechanism of action of the sulfotransferases has recently progressed by the discovery that the chondroitin 4-O sulfotransferase encoded by *CHST11* is involved in metastasis of breast cancer [15] and the chondroitin 4,6-O sulfotransferase encoded by *CHST15* in Lewis lung carcinoma (LCC) [16, 17]. However, Ust (small letters, because mouse) has not been studied in this context. Interestingly, B16 melanoma cells have 1.5 times more DS compared to LCC [18] suggesting that 2-O sulfation of CS/DS might play an important role in melanoma metastasis.

Previous reports showed that CS/DS affects cell adhesion and migration [7, 19] and that the lack of l-IdoUA on the cell surface leads to an impaired directed cell migration [20]. In the central nervous system, a tissue rich in CS-proteoglycans, over-sulfated CS are involved in neuronal migration and axon regeneration [19, 21]. Recently, a reduction in *CHST11* has been reported for siRNA-mediated versican knock-down in a leiomyosarcoma smooth muscle cell line [22]. Furthermore, the lack of Ust in skin of decorin-deficient mice impairs Fgf2 and Fgf7 binding and keratinocyte differentiation [23]. The occurrence of 2-O sulfated cell surface CS/DS can tune the Fgf2-mediated effect on cell migration of CHO cells and fibroblasts [5, 23].

A critical strep in migration is cell adhesion which is mainly mediated via integrins, heterodimeric cell surface receptors which mediate bidirectional signaling between cells and the extracellular matrix (ECM). During cell migration the function of α5β1 integrin and αvβ3 integrin is tightly regulated [24]. The role of α5 integrin in cancer progression is controversial [25]. α5 integrin also plays an important role in melanoma cell motility since its upregulation enhances migration [26, 27]. This is further supported by findings that human carcinomas frequently express high levels of α5β1 integrin which had been correlated with a more aggressive carcinoma phenotype [25]. For B16F10 melanoma cells a direct correlation of the metastatic potential and increased α5 integrin function was demonstrated [28].

The aim of the present study was to demonstrate that Ust is a critical regulator of melanoma cell adhesion and motility *in vitro* and *in vivo*. Reduced expression of Ust could be linked to a significant reduction of α5 integrin mRNA and protein in mouse and human melanoma cells. Our *in vivo* data showed that B16V^shUst(16)^ cells have a significantly reduced pulmonary metastatic potential. Therefore, we can link for the first time Ust and CS/DS 2-O sulfation with α5 integrin expression, an important factor for metastasis of melanoma cells.

## Materials and Methods

### Materials

The following primary antibodies were used: UST D-20 (Santa Cruz Biotechnology), β-actin, anti α5 integrin, anti β1 integrin (Millipore), Alexa Fluor^®^ 647 anti-mouse CD49e, LEAF^™^ β1, α5, αv and β3 integrin blocking antibodies (anti-mouse, BioLegend, California, USA) anti-rabbit-HRP secondary antibody (GE Healthcare, UK). F-actin was visualized by Alexa488-conjugated phalloidin (Invitrogen, USA). PD173074, fibronectin, mouse-Fgf2, chondroitin 6-sulfate (CS-6S) (Sigma Aldrich, Deisenhofen, Germany), chondroitin ABC lyase and heparitinase mix (heparinase II/III, 4:1) (Amsbio, UK).

### Cell culture

Murine melanoma (B16V) cells [29] were grown to confluence in bicarbonate buffered RPMI 1640 (Sigma) supplemented with 10% (v/v) bovine serum (FBS) at 37°C in a humidified atmosphere of 5% CO_2_. Of note, B16V cells display a black color due to their melanin. All experiments were performed at passages where cells contained melanin. Human HT168-M1, HT199 [30] and MV3 [31] melanoma cells were grown in RPMI 1640 with 10% (v/v) FBS and cultured as described before.

### Knock-down of *Ust* in melanoma cells

B16V cells were stably transfected with shRNA-Ust(m) plasmid as a pool of 3 target-specific lentiviral vector plasmids, each encoding 19–25 nt (plus hairpin) shRNAs designed to knock-down *Ust* gene expression (Santa Cruz), following the manufacturer’s protocol. Control cells were mock transfected with shRNA plasmid-A. Cells were selected with 10 μg/ml puromycin (Santa Cruz) for 2 weeks and further subcloned by single cell limiting-dilution. For human MV3 melanoma cells, UST siRNA and the respective scrambled siRNA were used according to the manufacturer (Santa Cruz) and the cells were analyzed 48 h after transfection.

### RNA extraction and quantitative real-time PCR

Cells were harvested using RNeasy Kit and RNA transcribed into cDNA using Omniscript RT Kit (both Qiagen, Germany) as described before [32]. cDNA corresponding to 25 ng of total RNA was used as a template. Expression levels of *Ust* (mouse and human), *β-actin*, *ubiquitin* (primer sequence: [23, 33]), *Itgb*1 (mItgb1-for 5`-CAA GAG GGC TGA AGA TTA CC-3`, mItgb1-rev 5`-GGC ATC ACA GTT TTA TCC A-3`), *Itgb*3 (mItgb3-for 5`-TGG TGC TCA GAT GAG ACT TTG TC-3`, mItgb3-rev 5`-GAC TCT GGA GCA CAA TTG TCC TT-3`), *Itga*5 (mItga5-for 5`-TGC TAC CTC TCC ACA GAA AAC-3`, mItga5-rev 5`-GCC AGT CTT GGT GAA CTC AG-3`), *ITGA*5 (hITGA5-for 5`-TGG CCT TCG GTT TAC AGT CC-3`, hITGA5-rev 5`- GGA GAG CCG AAA GGA AAC CA-3`), *Fgf*R1 (mFgfR1-for 5`-CAA CAA GAC AGT GGC CCT GGG-3`, mFgfR1-rev 5`-CCG TGC AAT AGA TAA TGA TC-3`) and *Fgf*R3(mFgfR2-rev 5`-CTC CAG ATA ATC TGG GGA AG3`, mFgfR3-for 5`- GGA GTT CCA CTG CAA GG-3`) were monitored by real-time PCR (ABI PRISM 7500, Applied Biosystems) using MESA GREEN qPCR Kit (Eurogentec, Germany). Raw data were normalized to the geometric mean of the control genes *β-actin* and *ubiquitin*. Two or more housekeeping genes lead to much more accurate results [34].

### Western blots analysis

~1x10^6^ melanoma cells were lysed using a lysis buffer (7 M Urea, 2 mM Thiourea, 40mM Tris-HCl, 0,001% (w/v) bromphenol blue, 1% (w/v) ASB-14). Cell lysates were cleared through a 0.2 μm filter, 20–40 μg of protein lysates were analyzed for Ust and α5 integrin. They were visualized with enhanced chemiluminescence (Perkin-Elmer Life Sciences, USA) and monitored with Fusion-SL 4.2 MP (PeqLab, Germany). Intensities were quantified as described previously [23, 33]. Of note, immune blots of the lysates before and after filtration led to the same results. The influence of the cell surface sulfation was evaluated after 6h of cell treatment with 30 mM NaClO_3_ [5]. For blocking FgfR, cells were incubated for 6h with PD173074 (20 mM) inhibitor as determined based on titration curves.

### Sulfotransferase activity of B16 cell lines

Sulfotransferase reaction was carried out according to the manufacturer’s instructions in a 96-well plate using the universal sulfotransferase assay (R&D). Briefly, protein lysates (25–200 μg) of B16V cell lines were incubated with 10 mM chondroitin 6-sulfate as substrate, PAPS (R&D), and a coupling phosphatase as control. The color was developed with a Malachite reagent for 20 min at room temperature and monitored at 620 nm with an ELISA reader. A phosphate standard curve was used to determine the activity (OD/pmol). The specific activity was determined with the following equation: Specific activity (pmol/min)/μg) = S(OD/μg) x CF(OD/pmol) / Time(min), where S is the slope of the line with the OD values of the sulfotransferase assay and CF the phosphate conversion factor (taken from the phosphate standard) [5].

### Characterization of cell surface chondroitin/dermatan sulfate and heparan sulfates

GAGs were extracted from ~2x10^7^ cells and highly-sulfated cell surface CS/DS were released by β-elimination and purified as described previously [23]. The HexUA content was determined using an m-hydroxydiphenyl reaction. Uronic acid was hydrolyzed in 80% sulfuric acid containing tetraborate at 80°C, incubated with m-hydroxydiphenyl (Sigma Aldrich) at room temperature and measured at 540nm using heparin as standard [5].

10 μg CS/DS were digested with 10 mU of chondroitin ABC for 2h. The unsaturated disaccharides were labeled with 5 μl of 0.1 M 2-Aminoacridon (AMAC) in 15% CH_3_COOH/DMSO solution. After 10 min incubation at RT, 1 M NaBH_3_CN was added and the mixture was incubated 16 h at 37°C followed by fluorophore assisted carbohydrate electrophoresis (FACE). AMAC-labeled disaccharides were separated on 30% Borate-polyacrylamid gel [35]. HS were analyzed as described before. In order to analyze HS composition, cell pellets of B16V cell lines were prepared as described previously. After enzymatic removal of CS/DS, the heparin lyase I-, II- and III- digested GAGs were fractioned by RPIP-HPLC. The peaks were identified by co-elution with standard HS disaccharides [5].

### Proliferation of melanoma cells

3×10^4^ B16V cells/cm^2^ cells were seeded and cultured for 24h and starved for 16h prior to the experiment. Experiments were performed in serum-free RPMI and proliferation was determined by BrdU incorporation for 16h (Cell Proliferation ELISA, Roche).

### Cell adhesion assay

Static adhesion assays were performed with 1×10^6^ cells of the different B16V cell lines or MV3 cells in the presence of the fluorescent marker 2′7′-bis-(2 carboxyethyl)-5 carboxyfluorescein acetoxy-methyl ester (Molecular Probes, USA) dissolved in DMSO as described previously [32, 36]. Labeled cells were seeded in non- or fibronectin-coated (10 μg/ml) 96-well plates and incubated for 30 to 360 min at 37°C. Cell adhesion to fibronectin was quantified after 1h with an ELISA reader (Epoch, Bioteck) as previously described [32]. For further adhesion experiments cells were preincubated with i) the different LEAF^™^ integrin blocking antibodies (5 μg/ml) for 3h at 37°C [33], ii) 6h pre-treatment with 30(mM NaClO_3_ or iii) enzymatic digestion of cell surface HS and CS/DS with 4 mU heparitinase II/III and/or chondroitin ABC lyase for 1h at 37°C.

### Wound scratch assay and migration on 3D collagen-rich matrices

1×10^5^ cells/cm^2^ of B16V cell lines were seeded in 12-well plates in RPMI medium and starved overnight. An artificial wound was generated and cells were incubated with serum-free RPMI medium (control) or RPMI supplemented with 10 ng/ml Fgf2 for 20h at 37°C. Images were captured at time points 0 and 20h, using a Zeiss Axiovert 100 microscope with AxioCam ICc1 camera. Cell migration was evaluated as described [5]. For each well 2–4 pictures were acquired (n = 3 independent experiments).

Primary *C57BL/6* skin fibroblasts were cultured for 10 days in 35 mm petri dishes with 1 mM L-ascorbate-2-phosphate (Sigma) to obtain a 3D ECM [37]. Confluent B16V cell lines were detached from the culture dish with trypsin/EDTA, and B16V cell suspension in serum-free RPMI1640 was added to the 10 day old and 24h serum-starved *C57BL/6* fibroblast cultures. Migration of cells was monitored, evaluated and calculated as described before [38].

### Immunofluorescence analysis

1.2×10^4^ cells/cm^2^ cells were seeded in 8-wells slides (Zell-Kontakt, Germany) and incubated for 24h. Cells were fixed with 4% PFA/PBS and then blocked with 3% BSA/PBS for 30 min. Cell surface α5 integrins were incubated with primary antibody Alexa Fluor^®^ 647 anti-mouse CD49e for 1h. Actin cytoskeleton and nuclei were co-visualized with phalloidin-Alexa-488 and DAPI, respectively. Fluorescence was monitored by a confocal microscope (Zeiss AxioImager M2) with 5–10 pictures per well (n = 3 independent experiments).

### Phospho-FGFR1 cell-based phosphorylation ELISA

Mouse/Human/Rat Phospho-FGFR1/FGF Receptor 1 (Y654) Cell-Based Phosphorylation ELISA Kit was used to determine the activation state of FgfR1 according to manufacturer instructions (LifeSpan Biosciences). Briefly, 20.000 cells/well were seeded in a 96-well plate and incubated overnight. Cells were starved overnight followed by treatment with PD173074 (20 nM) for 6 h to inhibit FgfR1. Cells were fixed with 4% PFA for 20 min, washed and blocked for 1 h prior to the incubation with the first antibodies i) anti-FGFR1-Phospho-Y654, ii) anti-FGFR1 or iii) anti-GAPDH overnight at 4°C. HRP-conjugated secondary antibody was incubated for 30 min and developed with a ready to use substrate. The enzyme activity was measured at OD450 nm (Epoch, Bioteck). GAPDH served as an internal positive control to normalize the values. Following the colorimetric measurement the crystal violet whole-cell staining method was used to determine cell density. After staining, the results were analyzed by normalizing the absorbance values to cell amounts. pY654-FgfR1 was normalized to FgfR1 and GAPDH. The same protocol was applied for α5 integrin and normalized to GAPDH and cell number.

### FACS analysis of cell surface α5 integrin

1 x 10^6^ of B16V cell lines were seeded in a 6-well plate for 24 h. Cells were washed with cold PBS, scraped from the plates and aliquoted to 1 x 10^6^ cells in 10 μl 2% FBS/PBS. To detect α5 integrin cells were incubated with Alexa Fluor^®^ 647 anti-mouse CD49e antibody (0.5 μg/100μl) for 30 min at 4°C. After 3 x washing with cold PBS, cells were resuspended in 1 ml 2% FBS/PBS and analyzed with FACSAria IIu. Non-stained and isotope controls were analyzed simultaneously.

### Animal experiments and B16 syngenic tumors

10 weeks old female C57BL/6 mice (Charles River, Germany) were grouped into 5 and kept for one week prior to the experiments and cared according to the standards of the German Council on Animal Care and Institutional Animal Care and Use Committee. Animals were housed in the animal facility of the Medical Faculty, University of Münster, Germany. Standard rodent chow and water were available *ad libitum* throughout the study and shredded paper was available for nest building. Mice were housed using a 14:10 light:dark cycle starting at 06:00 a.m. This study was carried out in strict accordance to the German Council on Animal Care under a specifically approved protocol by the ethics committee LANUV, NRW, Germany (protocol #84–02.04.2013.A007). All surgery was performed under isoflurane anesthesia, and all efforts were made to minimize suffering. 10^6^ cells parental control and B16V^shUst(16)^ cells in 70 μl PBS were injected s.c. into the right flanks of the mice. Mice and primary tumors were monitored every other day. Tumors were categorized as + < 0.5 cm^3^, ++ = 0.5–1 cm^3^, +++ > 1 cm^3^. Animals found with clinical signs, like weight loss or respiratory difficulty, were subjected to euthanasia. Euthanasia was carried out with an overdose of inhalant anesthetic followed by cervical dislocation. Primary tumors were removed after 15–21 days because of the size of the tumor (tumor size: B16V from 0.09 to 1.78 mg and B16V^shUst(16)^ from 0.1 to 3.9 mg) and weighed. Metastasis was monitored over a 6–7 week period followed by autopsies of the sacrificed animals [38]. Animals found with clinical signs were subjected to euthanasia. Pulmonary metastasis was evaluated macroscopically.

### Statistical analysis

Statistical evaluation was performed with GraphPad Prism4 using Student’s *t*-test. *P*<0.05 was considered as significant.

## Results

### Silencing *Ust* in B16V cells

To demonstrate that human cancer cells express UST we analyzed the human melanoma cell lines HT168-M1, HT199 and MV3 [29, 31] by qRT-PCR. All 3 cell lines express human *UST* (S1 Fig) with ΔCT ranging from ~1.67 to ~3.69. We previously published the *Ust* expression of CHO-K1 cells which showed a ΔCT value of ~3.4 [5].

**Fig 1:**
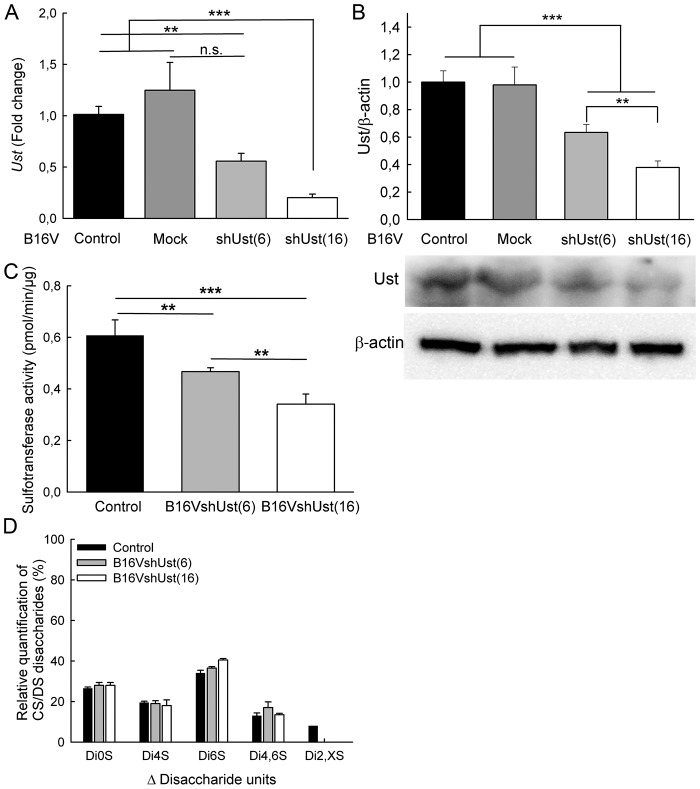
Modulation of Ust expression in melanoma cell lines. (*A*) Total RNA and cell lysates of B16V control, mock transfected B16V (B16V^mock^) and clones of B16V^shUst^ were analyzed by qRT-PCR. *Ust* expression was normalized to the housekeeping genes *β-actin* and *ubiquitin*. (*B*) Immunoblots of protein lysates were probed for Ust and β-actin of different transfected B16V melanoma cell lines (lower panel). Band intensities were quantified and Ust signals were normalized to β-actin (upper panel). (*C*) Sulfotransferase activity of the cell lines B16V, B16V^shUst(6)^ and B16V^shUst(16)^. (*D*) Highly-sulfated cell surface CS/DS disaccharide composition of the cell lines B16V, B16V^shUst(6)^ and B16V^shUst(16)^. Data shown are the mean±SEM (n≥4); **, *P*<0.01, ***, *P*<0.001).

B16V melanoma cells, which also express *Ust* mRNA (ΔCT of ~4) and protein (Fig 1A and 1B), have a highly metastatic potential *in vivo* [39]. To define the functional contribution of Ust to melanoma metastasis, lentiviral particles carrying shRNAs (shUst) were used to knock-down *Ust* in B16V cells. We also generated the respective mock controls. 20 clones were isolated and the *Ust* knock-down efficiency was determined. B16V and B16V^mock^ had similar Ust expression. Clone 6 (B16V^shUst(6)^) and 16 (B16V^shUst(16)^) showed a down-regulation of *Ust* mRNA by ~44% and ~80% (Fig 1A) and were further analyzed. Protein amounts revealed a reduction by ∼37% and ∼63%, respectively, for the two tested clones (Fig 1B; upper panel). Of note, B16V control and B16V^mock^ cells displayed no differences in the amount of Ust protein, so that B16V cells could be used as a control. Next, we determined sulfotransferase activity of cells. The substrate chondroitin 6-sulfate is converted to chondroitin 2,6 sulfate by Ust. B16V cells displayed a total sulfotransferase activity of 0.61±0.06 pmol/min/μg. Both B16V^shUst(6)^ (0.47±0.01 pmol/min/μg) and B16V^shUst(16)^ (0.34±0.04 pmol/min/μg) showed a significantly lower activity (Fig 1C) correlating with the *Ust* knock-down. FACE analysis of the GAGs after the sulfotransferase assay confirmed the reduced amount of ΔHexUA(2S)-GalNAc(6S) (ΔDi2,6S-units) for both cell clones (S2A and S2B Fig).

The reduced Ust enzyme activity affected also the 2-O sulfated units at the cell surface. Highly-sulfated CS/DS purified from *Ust* knock-down cells lack detectable amounts of 2-O sulfated disaccharide units compared to the control cells (Fig 1D). Of note, the uronic acid content of the highly-sulfated cell surface CS/DS was similar for all three tested cell lines (S2C Fig). For B16V we detected 5 different disaccharide units, for B16V^shUst(6)^ and B16V^shUst(16)^ only 4 different disaccharide units. The percentages of ΔHexUA-GalNAc (ΔDi0S) and ΔHexUA-GalNAc(4S) (ΔDi4S) were similar in all three cell lines (Fig 1D). ΔHexUA-GalNAc(6S) (ΔDi6S) and ΔHexUA-GalNAc(4S,6S) (ΔDi4,6S) units displayed slight alterations. Interestingly, we did not detect any mono-sulfated ΔDi2S-units in B16V cells. B16V cells contained ~8±0.8% of di-sulfated ΔHexUA(2S)-GalNAc(4S or 6S) (ΔDi2,XS-units (X = 4 or 6)). HPLC analysis of total GAGs confirmed that the amount of ΔDi4S in the total CS/DS did not vary between the cell lines, indicating a similar amount of DS (S2D Fig). Furthermore, the ΔDi2,XS detected by FACE was confirmed by HPLC as ΔDi2,4S. As expected, a ∼63% Ust protein reduction in B16V^shUst(16)^ abolished 2-O sulfated units (Fig 1D). For ∼37% reduction of Ust we could not detect 2-O sulfated units by FACE. This can be explained by the limit of detection because the enzyme activity test showed also a ~40% less 2,6-O sulfation for B16V^shUst(6)^ cells (S2A and S2B Fig). HS analysis revealed no alterations in the disaccharide composition (S2E Fig). These data show that we generated B16V melanoma cell lines with different levels of Ust and 2-O sulfated CS/DS GAGs on the cell surface.

### Functional characterization of the B16V^shUst^ cells in vitro

**Fig 2:**
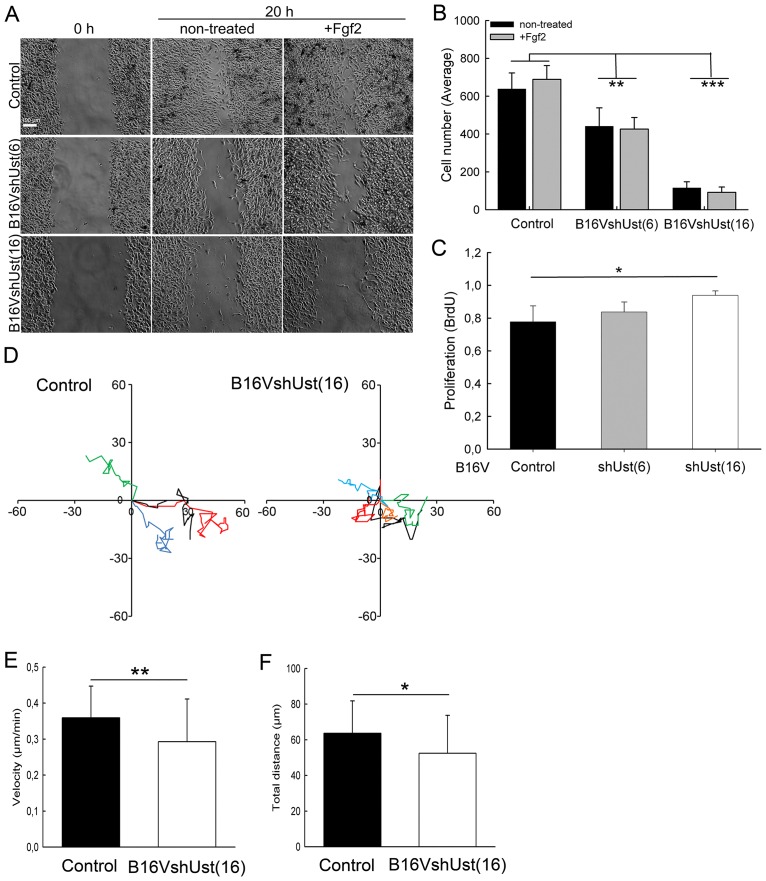
Migration of B16 melanoma cell lines. (*A*) Scratch assays were performed on B16V control, B16V^shUst(6)^ and B16V^shUst(16)^. Confluent cells were starved and wounded prior to Fgf2 treatment. Representative pictures are shown for 0 and 20h (Bar = 100 μm). (*B*) Quantification of the wound scratch assay shown in (*A*). Data are expressed as a mean±SD of three independent experiments (n = 8 for each condition). (*C*) Proliferation of the B16V cell lines measured by BrdU incorporation for 20h. (*D*) Paths of four migrating cells of B16V control and B16V^shUst(16)^ evaluated by time lapse-microscopy. (*E*) Quantifications of the migration of control and B16V^shUst(16)^ velocity (Supporting Information S3 and S4 Figs) and (*F*) total distance covered on murine wild-type fibroblast matrices (three independent experiments, mean±SD, 32 control cells and 54 B16V^shUst(16)^ cells, *, *P*<0.05, **, *P*<0.01, ***, *P*<0.001).

Previously, the importance of cell surface DS [20] and 2-O sulfation [5, 23] for cell migration was reported. In the present study, scratch assays showed that B16V melanoma cells close the gap within 20h (Fig 2A). The reduction of Ust led to a significantly slower migration of B16V^shUst(6)^ and B16V^shUst(16)^ cells on plastic. Fgf2 is a critical regulator of melanoma progression and it is expressed by melanoma cells [40]. However, Fgf2 addition had no impact on cell migration (Fig 2A and 2B). To exclude an overlap of migration and proliferation, BrdU incorporation was assessed. Within 24h under serum-free conditions B16V and B16V^shUst(6)^ cells showed similar proliferation rates, only B16V^shUst(16)^ cells displayed a significant increase in proliferation (Fig 2C). Therefore, we can conclude, that proliferation does not interfere with migration. Since molecular analysis of clone 6 showed no significant but detectable changes, we mostly show clone 16 in the following.

To get closer to an *in vivo* situation we used skin fibroblasts which were cultured for 10 days to deposit their own 3D ECM. Migration of B16V cell lines was monitored by time-lapse microscopy (movies S3 and S4 Figs). Tracking single cells on the ECM showed that B16V^shUst(16)^ cells migrated significantly more slowly (0.30±0.12 μm/min) than controls (0.35±0.09 μm/min) (Fig 2D and 2E). Furthermore, controls covered significantly longer distances than B16V^shUst(16)^ cells (Fig 2F).

These results show that Ust and consequently CS/DS 2-O sulfation affect melanoma cell migration. To clarify the altered migration of the B16V^shUst^ cell lines we analyzed cell adhesion.

### Lack of Ust in B16V affects adhesion to fibronectin

**Fig 3:**
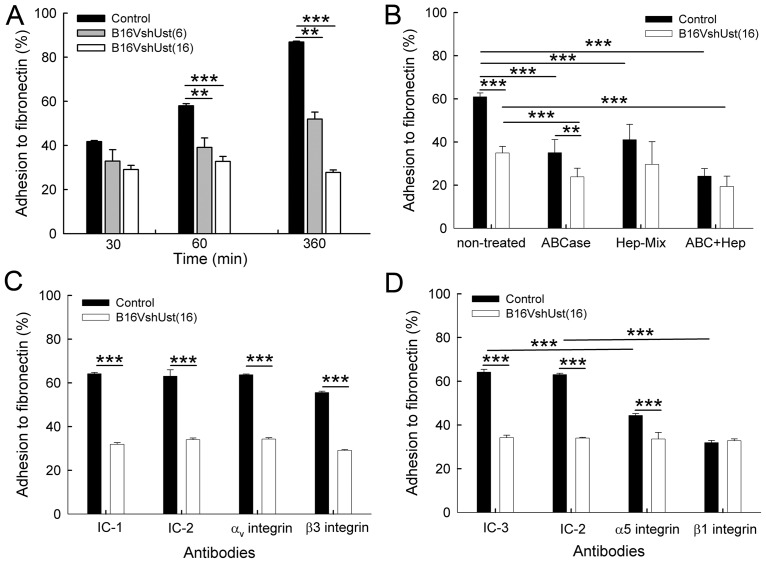
Adhesion of the B16V melanoma cell lines to fibronectin. (*A*) Adhesion of B16V control, B16V^shUst(6)^ and B16V^shUst(16)^ cells in fibronectin-coated wells. (*B*) Adhesion of B16V control and B16V^shUst(16)^ cells to fibronectin after 1h after treatment with chondroitin ABC layse (ABCase), heparitinase (Hep-Mix) and ABCase+Hep-Mix. (*C*) Adhesion of B16V and B16V^shUst(16)^ cells to fibronectin for 1h after blocking of αvβ3 integrin. The integrins were blocked with the αv integrin blocking antibody, isotype control IC-1 (Rat IgG1, κ as control against αv integrin), β3 integrin blocking antibody and isotype control IC-2 (Armenian Hamster IgG towards β3 integrin). (*D*) Adhesion of B16V and B16V^shUst(16)^ cells to fibronectin after blocking with α5 integrin blocking antibody, isotype control IC-3 (Rat IgG2a, κ as control for α5 integrin) and β1 integrin blocking antibody and isotype control IC-2 (Armenian Hamster IgG for β1 integrin). Each experiment was performed in duplicates, n = 3, mean±SD (*A*, *B*), mean±SEM (*C*, *D*), *, *P*<0.05, **, *P*<0.01, ***, *P*<0.001).

Cell surface GAGs influence adhesion and migration of cancer cells [41]. Assessment of B16V^shUst^ cell adhesion to plastic showed a significant reduction when compared to control (S5A Fig). Integrins, such as α5β1, are involved in adhesion to fibronectin [25]. After 1h and 6h, B16V cell adhesion to fibronectin was significantly higher compared to the B16V^shUst^ cell lines (Fig 3A). To demonstrate the influence of sulfation, cells were pre-treated with 30 mM NaClO_3_. Chlorate treatment leads to an inhibition of the 3’-phosphadenosine-5-phosphosulfate synthesis and reduction of the sulfate content of cell surface GAG [42]. Of note, after chlorate treatment B16V^shUst(16)^ cell adhesion monitored at the time point 1h was significantly lower compared to B16V cells (S5B Fig). To narrow down the type of GAGs involved in adhesion, cell surface CS/DS was digested with chondroitin ABC lyase and HS with heparitinase [5]. Enzymatic digestion of CS/DS or HS led to a significantly reduced adhesion of B16V cells compared to the non-treated cells after 1h. Upon digestion of cell surface CS/DS (ABCase), but not HS, we observed a significant difference between control and B16V^shUst(16)^ cells. Digestion of both CS/DS (ABCase) and HS (Hep-Mix) reduced adhesion of both cell types (Fig 3B). To determine which integrin dimer is responsible for the impaired adhesion of the B16V^shUst(16)^ cells, we used specific blocking antibodies for αvβ3 and α5β1 integrin and the respective isotype controls. Blocking of αv or β3 integrin had no impact on the adhesion of either B16V cells or B16V^shUst(16)^ cells to fibronectin when cells were compared to their isotype treated controls or the non-treated cells (Fig 3C). In contrast, blocking of α5 or β1 integrin significantly reduced adhesion of B16V to fibronectin compared to isotype treated controls or the non-treated cells (Fig 3D). Interestingly, B16V^shUst(16)^ cells showed still basal adhesion compared to isotopic treated control or the non-treated cells (Fig 3D). The results indicate that α5β1 integrin, but not αvβ3 integrin, mediates adhesion of B16V cells to fibronectin.

### *Ust* knock-down influences *Itga*5 and *FgfR*1 expression in melanoma cells

**Fig 4:**
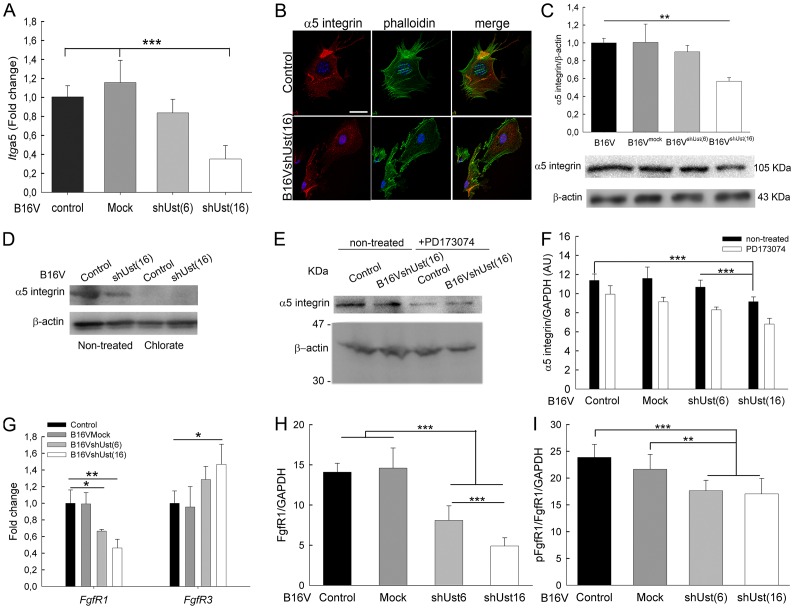
α5 integrin expression in murine B16 cells. (*A*) B16V cell lines analyzed for *Itga*5 expression by qRT-PCR. *Itga*5 expression was normalized to the housekeeping genes *β-actin* and *ubiquitin*. (*B*) Distribution of α5 integrin on control and B16V^shUst(16)^ cells seeded on fibronectin for 3 h. α5 integrin (red), F-actin (green) and nuclei stained with DAPI (blue). Bar = 25 μm. Protein extracts of control, B16V^mock^, B16V^shUst(6)^ and B16V^shUst(16)^ cell lines were subjected to immuno blotting. (*C*) Immunoblots were performed to detect α5 integrin and β-actin (lower panel). Signal intensities were normalized to the loading control β-actin (upper panel) (n = 3–5 mean±SEM, **, *P*<0.01). (*D*) B16V control and B16V^shUst(16)^ cells were starved overnight and treated with 30 mM NaClO_3_ for 6h. Protein lysates were analyzed for α5 integrin and the loading control β-actin (lower panel). (*E*) Control and B16V^shUst(16)^ cells were starved overnight and treated with 20 mM PD173074 for 6h. Protein extracts were subjected to immuno blotting for α5 integrin and β-actin. (*F*) Quantification of α5 integrin after PD173074 treatment was obtained by ELISA with GAPDH as control. (*G*) B16V cell lines were analyzed for *Fgf*R1 and *Fgf*R3 expression by qRT-PCR and normalized to *β-actin* and *ubiquitin*. (*H*, *I*) B16V cell lines were analyzed for FgfR1 and pY654FgfR1 by ELISA. Fgfr1 was normalized to GAPDH and to total cell number. Data are shown as mean±SD (n = 3, *, *P*<0.05**, *P*<0.01, ***, *P*<0.001).

To investigate the connection of CS/DS 2-O sulfation and α5 integrin-mediated adhesion *Itga*5 expression was analyzed. For control and mock transfected B16V cells *Itga*5 expression was similar. B16V^shUst(6)^ (-20%) and B16V^shUst(16)^ cells displayed a reduction, however, only B16V^shUst(16)^ cells expressed significantly less *Itga*5 (Fig 4A). Surprisingly, *Igtb1* and *Igtb3* expression was increased in B16V^shUst^ cells (Table 1).

**Table 1:** Expression of integrins in B16 cell lines.

Gene	Protein	Mock	B16V^shUst(6)^	B16V^shUst(16)^
*Itgb1*	β1 integrin	1.43 ± 0.37	2.28 ± 0.32	3.6 ± 0.5
*Itgb3*	β3 integrin	1.37 ± 0.25	2.23 ± 0.18	2.64 ± 0.37

B16V, B16V^mock^, B16V^shUst(6)^ and B16V^shUst(16)^ cells were analyzed for *Itgb1* and *Itgb3* expression by qRT-PCR. Expression was calculated as described in Materials & Methods (n = 6) and normalized to parental B16V cells. *Itgb1* expression levels are significantly increased in B16V^shUst(16)^ cells compared to B16V^mock^ cells. Data are presented as the Fold-change±SEM.

Control and B16V^shUst(16)^ cells were seeded on fibronectin and analyzed by confocal microscopy. F-actin and the distribution of α5 integrin were evaluated after 3h (Fig 4B). In contrast to control, B16V^shUst(16)^ cells showed less α5 integrin and altered F-actin distribution which might explain the impaired adhesion of the *Ust* knock-down cells (Fig 3B). The immunofluorescence results were supported by immunoblots for α5 integrin. Controls showed similar amounts of α5 integrin whereas for B16V^shUst(16)^ cells α5 integrin was significantly reduced (Fig 4C). Again, B16V^shUst(6)^ showed also a reduced amount of α5 integrin similar to the qRT-PCR data. Next, we determined the amount of α5 integrin on the cell surface by FACS analysis and observed no differences for all tested cell lines (S6A, S6B and S6C Fig). To link GAG sulfation to integrins the cells were treated with chlorate. Chlorate treatment depleted α5 integrin in both cell lines (Fig 4D), indicating a link between sulfation and α5 integrin. *Itga*5 is known to be regulated by Fgf2 [43, 44]. Therefore, we used the specific inhibitor PD173074 to target FgfR1. Blocking FgfR1 for 6h led to a reduction of α5 integrin of control, mock, B16V^shUst(6)^ and B16V^shUst(16)^ cells (Fig 4E and 4F). Next, we analyzed *FgfR*s transcripts and only *FgfR*1 and 3 showed higher expression levels. Interestingly, the expression levels of *FgfR*1 in the B16V cell lines were significantly reduced correlating with the decrease of Ust. *FgfR*3 was significantly increased only in the B16V^shUst(16)^ cells (Fig 4G).

Next we determined the amount of FgfR1 and its activation in the B16 cell lines. An ELISA type assay showed a significant reduction of FgfR1 protein in the knock-down cells correlating with the amount of Ust (Fig 4H). Moreover, the activation of FgfR1-Y654 showed that both knock-down cells lines displayed significantly less phosphorylation compared to control and mock transfected B16V cells (Fig 4I). Therefore, we can conclude that, depending on Ust levels, *ITGa*5 and *FgfR*1 expression is affected as well as the activation of FgfR1. In addition, 2-O sulfated CS/DS proteoglycans influence the function of α5β1 integrin and consequently cell adhesion to fibronectin.

### Melanoma cell lung metastasis is influenced by *Ust* expression

**Fig 5:**
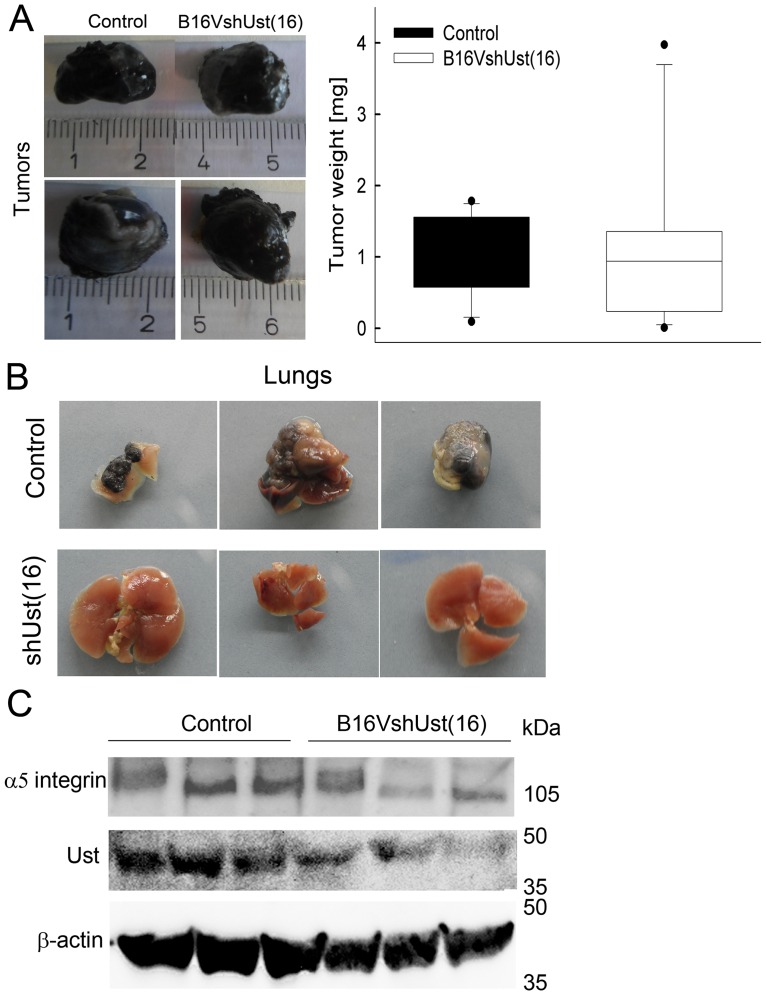
Pulmonary metastasis of B16V cells and analysis of the primary tumors. (*A*) Dissected control and B16V^shUst(16)^ primary tumors (left panel) and their weight (right panel) measured after 15–21 days of inoculation (three independent experiments n = 13–15). (*B*) Macroscopic evaluation of lungs of mice after 6–7 weeks of primary tumor dissection. Three representative pictures of each control and B16V^shUst(16)^ inoculated mice. (*C*) Representative blots of control and B16V^shUst(16)^ primary tumor lysates for Ust, α5 integrin and β-actin as loading controls.

The metastatic potential is greatly affected by the expression levels of integrins. Therefore, we investigated the behavior of B16V^shUst(16)^ cells *in vivo*. When control and B16V^shUst(16)^ cells were inoculated into female syngenic C57BL/6 mice no difference in tumor growth was observed (Fig 5A; Table 2; n = 12 for control and 11 for B16V^shUst(16)^). 15–21 days after the resection of the primary tumor, mice were sacrificed and further analyzed. There was no difference in weight of the mice (control: 21.36±2.1 g, B16V^shUst(16)^ 22.54±1.0 g). Macroscopic evaluation of lungs showed metastases in 6 out of 9 mice inoculated with control cells, whereas the 11 mice with B16V^shUst(16)^ tumors showed no macroscopic lung metastasis (Table 2 and Fig 5B). Notably, detection of lung metastasis was independent of the tumor size. Western blot analysis revealed a reduction of Ust and α5 integrin in the B16V^shUst(16)^ tumors (Fig 5C). The amount of β1 integrin in the tumor lysates was not altered (S7 Fig). These *in vivo* results support the *in vitro* observation of impaired B16V^shUst(16)^ adhesion due to a reduced amount of α5β1 integrin induced by the lack of Ust.

**Table 2:** *In vivo* experiments using control and B16V^shUst(16)^ cells in a C57BL/6 mice tumor metastasis model.

Experiment	Mice per strain	Removal of primary tumor		Analyzed after 6–7 weeks		Lung metastasis^$^	
		Con	B16V^shUst(16)^	Con	B16V^shUst(16)^	Con	B16V^shUst(16)^
V1	5	3 (2^+^)	3 (2^+^)	2 (1^§^)	3	1	0
V2	5	5	4 (1^#^)	3 (2^§^)	4	3	0
V3	5	4 (1*)	4 (1*)	4	4	2	0
Total	15	12	11	9	11	6	0

10^6^ cells were injected and after 15–21 days primary tumors were removed. After 6–7 weeks mice were dissected and macroscopically evaluated for lung metastasis.

^+^ mice died before tumor dissection

^#^ mice no tumor developed

* mice died during tumor dissection

^§^ mice died during 7 weeks

^$^ macroscopic evaluation

### *UST* in human melanoma MV3 cells

**Fig 6:**
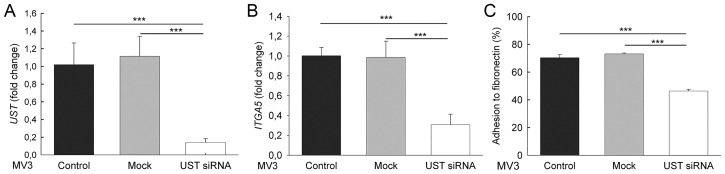
Human melanoma cells and *UST* knock-down. Expression levels of *UST* (*A*) and *ITGA*5 (*B*) in human MV3 melanoma cells after transient *UST* knock-down with siRNA. Expression was normalized to the housekeeping gene *β-actin*. (*C*) Adhesion to fibronectin of *UST* knock-down MV3 cells and the respective control. Data are shown as mean±SEM (n = 3, ***, *P*<0.001).

Our concept was supported by the results obtained with siRNA-mediated *UST* knock-down in human MV melanoma cells. *UST* siRNA transfection of MV cells caused an 80% reduction of UST mRNA (Fig 6A). In these cells *ITGa*5 was also reduced by 70% (Fig 6B), and consequently adhesion to fibronectin (Fig 6C). These results show that CS/DS 2-O sulfation mediates α5 integrin expression via FgfR at least after *Ust* knock-down.

**Fig 7:**
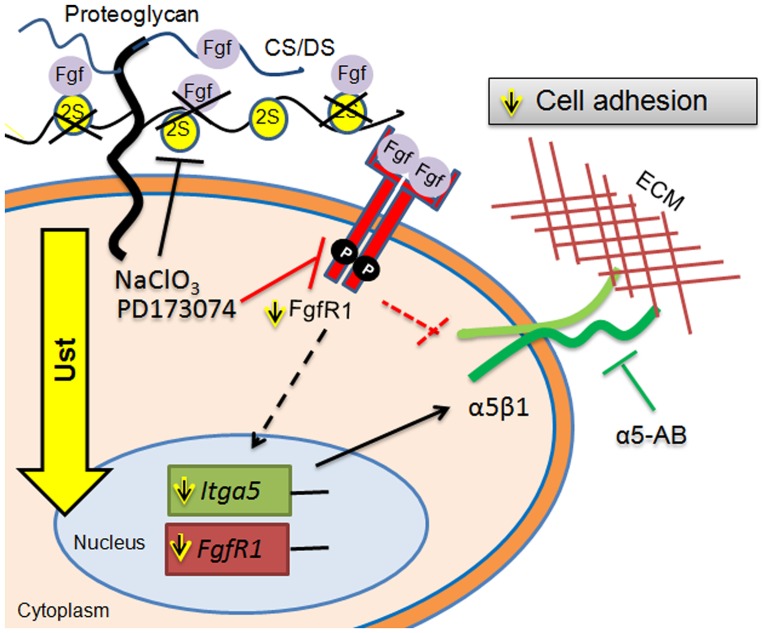
Model depicting the potential role of Ust and CS/DS 2-O sulfation in melanoma metastasis. Ust knock-down reduces 2-O sulfation of CS/DS proteoglycans and affects *Itga*5 expression possibly via FgfR1. The consequence of reduced sulfation is an impaired adhesion. α5-AB: blocking antibody for α5 integrin. For additional details refer to the text.

Our results show that Ust and 2-O sulfation levels of CS/DS affect the synthesis of *Itga*5 and *FgfR*1 and, in addition, the function of α5β1 integrin which leads to impaired melanoma cell adhesion (Fig 7).

## Discussion

Migration of tumor cells is an important step during metastasis. The motility of melanoma cells contributes to their highly invasive and metastatic potential. Melanoma cells display a shift from HS and DS to CS [13]. However, the function of the fine structure of the 2-O sulfation of the CS/DS chains and the respective enzymes involved, especially Ust, are not known. So far the function of Ust has been studied during brain development or under physiological conditions [5, 19]. CHO-K1 cells express Ust which subsequently leads to CS/DS 2-O sulfation, later involved in Fgf-2-induced migration [5]. Recently, a patient with a microdeletion on chromosome 6q25.1 was described with among other symptoms an Ehlers-Danlos syndrome in skin [45]. The microdeletion included the lack of human UST gene indicating that the minor sulfation of CS/DS affects also the organization of the extracellular matrix, similar to the DS [46]. Understanding the impact of Ust and 2-O sulfation might identify potential therapeutic targets in melanoma metastasis.

We used an experimental metastatic model of B16 cells which has the advantage of forming a primary orthologue tumor followed by metastasis from the skin to the lung [39]. Our *in vivo* experiments show a marked inhibition of pulmonary metastasis after the knock-down of *Ust* in B16V cells. The importance of the CS/DS fine structure has been shown in the LLC pulmonary metastasis model, were the knock-down of *Chst*15 reduced ΔDi4,6S units and consequently the transmigration of cells from the blood into the lung tissue [17]. A reduced proliferation was observed for the LCC *Chst*15 knock-down cells [17] or when DS was removed from melanoma cells [14]. In contrast, *in vitro Ust* knock-down increased proliferation in CHO-K1 [5] and B16V cells. *In vivo* we observed a similar size of the tumors for the B16V model which could be explained by similar amounts of ΔDi4,6S units. Of note, B16 cells contain 1.5 times more DS than LCC cells [18] indicating that adhesion and migration of melanoma cells could be influenced by 2-O sulfated CS/DS. Under physiological conditions, Ust has been shown to be important for *in vivo* cell migration and possibly development [19, 45].

The structures of GAGs influence migration, too. Aortic smooth muscle cells with reduced DS show a decrease in directional migration, although the velocity and the total distance are increased [20]. Of note, the reduction of DS in mice reduced also the 2-O sulfation of CS/DS [47]. Knock-down of DS-epimerase 1 and consequently DS in cancer cells also reduced migration [7]. Vice versa, CHO-K1 cells migrate faster when they overexpress Ust. B16V melanoma cells are highly metastatic [39] and display an increased velocity compared to CHO-K1 cells [5]. *Ust* knock-down in B16V cells consequently reduced migration, like *Chst*15 knock-down in LCC cells [17]. Our observation that the amount of ΔDi4S-units was not affected and migration was opposed to smooth muscle cells with reduced DS might indicate that the amount of DS on the cell surface of B16V cell lines is not affected by the *Ust* knock-down.

*Ust* knock-down in CHO-K1 cells and fibroblasts also results in an impaired migration as previously reported for neuronal outgrowth [5, 19, 23]. The residual migration of B16V^shUst(6)^ cells, in contrast to B16V^shUst(16)^, can be explained by the presence of ΔDi4,6S units [17] and the impact of the level of 2-O sulfation. *In vivo*, for HS not only the amount of sulfation but also the HS structure affects hedgehog signaling during development [48]. Migration on a complex ECM generated by fibroblasts requires integrins that recognize several matrix ligands including fibronectin (e.g. α5β1, αvβ3, α4β1) or collagens (α1β1, α2β1). Previously, we showed that the lack of decorin and the reduced amount of 2-O sulfated CS/DS in fibroblasts lead to an increase in β1 integrin [23, 33]. Melanoma cells express α5β1 and αvβ3 integrin which are involved in adhesion and migration [26] and are tightly regulated [24]. Overexpression of miR-148b in melanoma cells significantly inhibited metastasis by reducing *ITGa*5 [49]. In contrast to decorin-deficient fibroblasts, B16V *Ust* knock-down cells displayed a reduction in α5 integrin. B16V cell adhesion to fibronectin was only reduced by blocking α5β1 but not αvβ3. The cell surface amount of α5 integrin was not altered in B16V *Ust* knock-down cells indicating that 2-O CS/DS sulfation functions as a structural component involved in adhesion. Various studies showed that cell surface CS and DS and their structures have an impact on cancer cell adhesion [7, 14, 15, 17, 41]. One might speculate that the level of CS/DS 2-O sulfation on the cell surface modulates α5β1 integrin conformation and fibronectin binding. This speculation can be supported by a recent publication about different distinct global conformations of α5β1 integrin which determine adhesive and non-adhesive function to fibronectin. Under conditions in which the bent-closed conformation predominates, α5β1 integrin impairs adhesion to fibronectin in a K562 cell line [50].

As modulating signaling events, *Ust* knock-down and consequently, CS/DS 2-O sulfation are associated with the expression of *Itg*a5 and *FgfR*1. The reduced expression of *Itg*a5 and *FgfR*1 can be explained by the function of CS/DS as low affinity receptors for different growth factors [1, 2, 7] and therefore affecting signaling. This hypothesis is supported by a reduced *Itg*a5 expression either after inhibition of cell surface sulfation or by blocking FgfR1. Moreover, also FgfR1 and its activation are reduced in B16 *Ust* knock-down cells. The link of α5 integrin, Fgf2 and FgfR has been demonstrated for angiogenesis [43] and in 3T3 fibroblasts [44]. In addition, the aggressiveness of melanoma is due to Fgf2 induced α5 integrin expression [28]. Under physiological conditions Fgf2 signaling requires GAGs [1] and we could show that 2-O sulfated CS/DS are involved in migration [5]. A possible downstream mechanism could be the transcription factor Twist-1 which has been recently shown to induce *Itg*a5 expression and leads to epithelial-mesenchymal transition [51]. The link between reduced Twist-1 expression, lack of DS and adhesion to fibronectin has been shown for *Xenopus* neural crest cells [52]. Of note, adhesion of B16V^shUst(16)^ cells was significantly reduced by either inhibiting CS/DS sulfation or by digesting cell surface GAGs.

To support a possible role of Ust in melanoma, we tested three metastasizing human melanoma cell lines, MV3 [31], HT199 and HT168M [30] that all express *UST*. The biological relevance of the data obtained with mouse melanoma cells is supported by the human melanoma cell line MV3 which expresses *UST*. siRNA-mediated *UST* knock-down in MV3 cells also showed a reduction in *ITGa*5 and adhesion.

Overall our data propose Ust and consequently 2-O sulfated CS/DS as a regulator of adhesion via the amount and activation of FgfR1 and the expression of *Itga*5 in melanoma cells (Fig 7). In addition, the amount of 2-O sulfated CS/DS influences α5β1 integrin function in melanoma cells indicating that Ust could be a potential marker for melanoma metastasis and a target for a therapeutic approach.

## Supporting Information

S1 Fig *UST* expression in melanoma cell lines. qRT-PCR for *UST* of three human melanoma cell lines with high metastasizing potential and murine B16V cells. HT168-M1, HT199 (Ladányi et al., 2001) and MV3 cells (van Muijen et al., 1991) were previously described. HT168-M and HT199 revealed similar metastatic potential after intra-splenic injection (Ladányi et al., 2001). All tested cell lines express *UST*. ΔCT values show that all three human cell lines express more *UST* compared to B16V cells.
(JPG)

S2 Fig Characterization of B16^shUst^ cell lines. B16 cell lysates were subjected to the sulfotransferase assay (see Materials and Methods) followed by disaccharide analysis by FACE. CS6S was used as a substrate to determine the sulfotransferase activity and to obtain ΔDi2,6S units. The gel following FACE does not allow to distinguish between ΔDi2,6S and ΔDi2,4S therefore, we used ΔDi2,XS. (*A*) Borate gel shows a reduced amount of ΔDi2,XS in both B16V^shUst^ cell lines indicating a reduction in 2-O sulfotransferase activity due to the *Ust* knock-down. (*B*) The quantification of the signals (panel A) shows 40% less 2-O sulfated disaccharides for B16VshUst(6) and 70% less for B16VshUst(6). The FACE analysis supported the result obtained by the enzyme activity test (see Fig 1C). (*C*) Uronic acid content of the three B16V cell lines (n = 3). (*D*) Quantification of 4-sulfated disaccharides (ΔDi4S) derived from total cell surface CS/DS and (*E*) HS disaccharide analysis of B16V and B16VshUst(16) cells (n = 3).
(TIF)

S3 Fig Movie of the migration of control B16V cells (*B*) on 3D matrices generated by fibroblasts over 10 days. To obtain a collagen-rich ECM fibroblasts were cultured in the presence of ascorbate-2-phosphate. The time-lapse microscope took images in 5 min intervals for 2h.
(MOV)

S4 Fig Movie of the migration of B16^shUst(16)^ cells on 3D matrices generated by fibroblasts over 10 days. To obtain a collagen-rich ECM fibroblasts were cultured in the presence of ascorbate-2-phosphate. The time-lapse microscope took images in 5 min intervals for 2h.
(MOV)

S5 Fig Adhesion of control B16V and B16V^shUst(16)^ cells. (*A*) Time course for the cell adhesion to plastic. (*B*) Cell adhesion for 1 h to fibronectin after treatment with 30 mM chlorate for 6 h to inhibit GAG sulfation. Both regiments lead to a reduction of adhesion of the B16V cells to basal levels of B16V^shUst(16)^ cells, indicating that CS/DS sulfation is involved in adhesion to fibronectin.
(TIF)

S6 Fig Cell surface α5 integrin determined by FACS. Histogram of cell surface α5 integrin expression in B16V, B16V^mock^, B16V^shUst(6)^ and B16V^shUst(16)^ cell lines. Living cells were stained with (*A*) the antibody CD49e-Alexa647 or (*B*) the isotype control and subjected to FACS analysis. (*C*) Unstained cells were used as control. The histograms are one out of three representative experiments and display the same amount of α5 integrin on the cell surface of the 4 cell lines (n = 3).
(TIF)

S7 Fig Detection of β1 integrin in the tumors. Immuno blots of three control and three B16V^shUst(16)^ primary tumors lysates for β1 integrin and β-actin as loading control. The β1 integrin blot was used after stripping. Therefore, the loading control β-actin is the same as in Fig 5C.
(TIF)

## Abbreviations

B16V: Mouse melanoma cell line
CS/DS: chondroitin/dermatan sulfate
GAG: glycosaminoglycan
MV3: human melanoma cell line
Ust: uronyl 2-O sulfotransferase
