# Supplementary figures and images for: Melanoma Cell Adhesion and Migration Is Modulated by the Uronyl 2-O Sulfotransferase
Source: PLoS One. 2017 Jan 20;12(1):e0170054. doi: 10.1371/journal.pone.0170054 (PMC5249195; doi:10.1371/journal.pone.0170054)

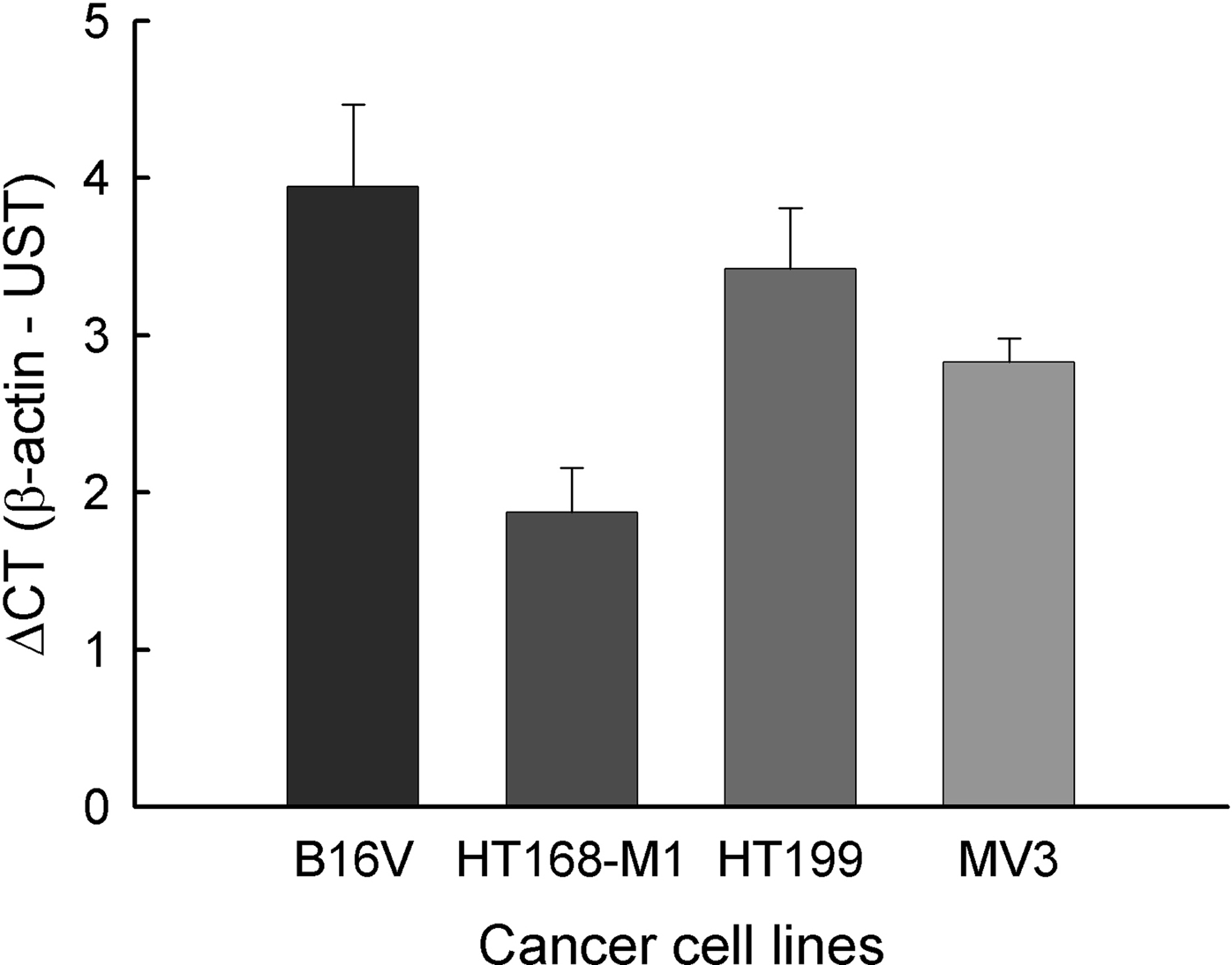

Supplement: S1 Fig — qRT-PCR for UST of three human melanoma cell lines with high metastasizing potential and murine B16V cells. HT168-M1, HT199 (Ladányi et al., 2001) and MV3 cells (van Muijen et al., 1991) were previously described. HT168-M and HT199 revealed similar metastatic potential after intra-splenic injection (Ladányi et al., 2001). All tested cell lines express UST. ΔCT values show that all three human cell lines express more UST compared to B16V cells. (JPG) [file pone.0170054.s001.jpg]

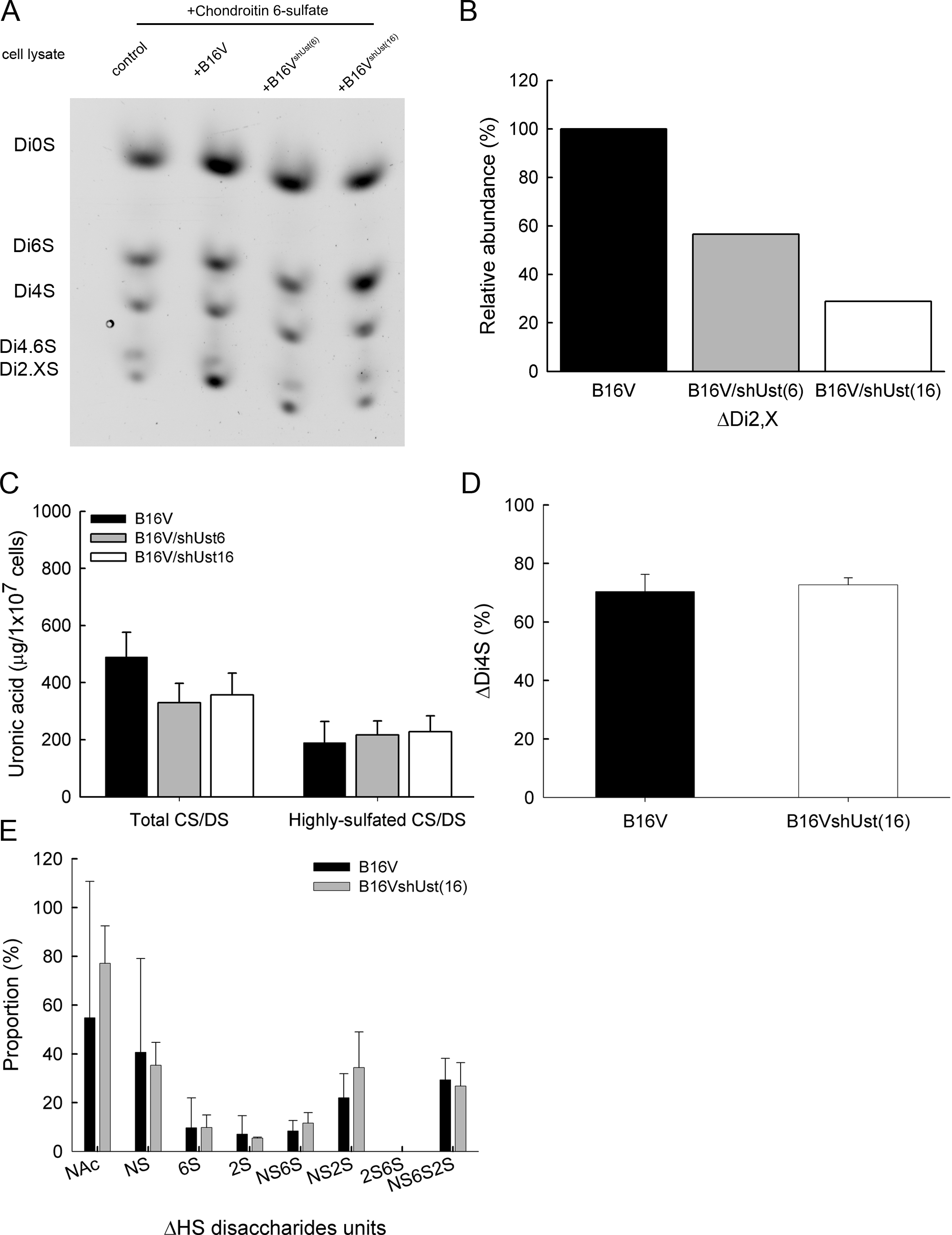

Supplement: S2 Fig — B16 cell lysates were subjected to the sulfotransferase assay (see Materials and Methods) followed by disaccharide analysis by FACE. CS6S was used as a substrate to determine the sulfotransferase activity and to obtain ΔDi2,6S units. The gel following FACE does not allow to distinguish between ΔDi2,6S and ΔDi2,4S therefore, we used ΔDi2,XS. (A) Borate gel shows a reduced amount of ΔDi2,XS in both B16VshUst cell lines indicating a reduction in 2-O sulfotransferase activity due to the Ust knock-down. (B) The quantification of the signals (panel A) shows 40% less 2-O sulfated disaccharides for B16VshUst(6) and 70% less for B16VshUst(6). The FACE analysis supported the result obtained by the enzyme activity test (see Fig 1C). (C) Uronic acid content of the three B16V cell lines (n = 3). (D) Quantification of 4-sulfated disaccharides (ΔDi4S) derived from total cell surface CS/DS and (E) HS disaccharide analysis of B16V and B16VshUst(16) cells (n = 3). (TIF) [file pone.0170054.s002.tif]

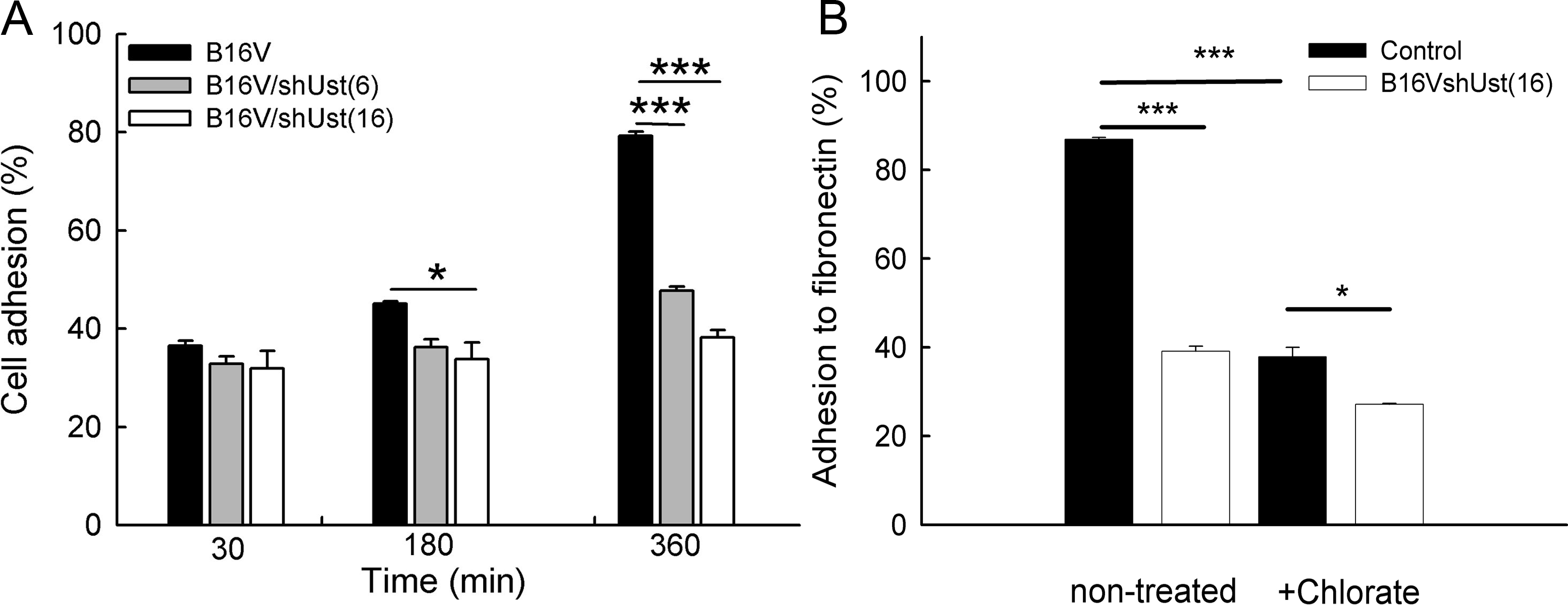

Supplement: S5 Fig — (A) Time course for the cell adhesion to plastic. (B) Cell adhesion for 1 h to fibronectin after treatment with 30 mM chlorate for 6 h to inhibit GAG sulfation. Both regiments lead to a reduction of adhesion of the B16V cells to basal levels of B16VshUst(16) cells, indicating that CS/DS sulfation is involved in adhesion to fibronectin. (TIF) [file pone.0170054.s005.tif]

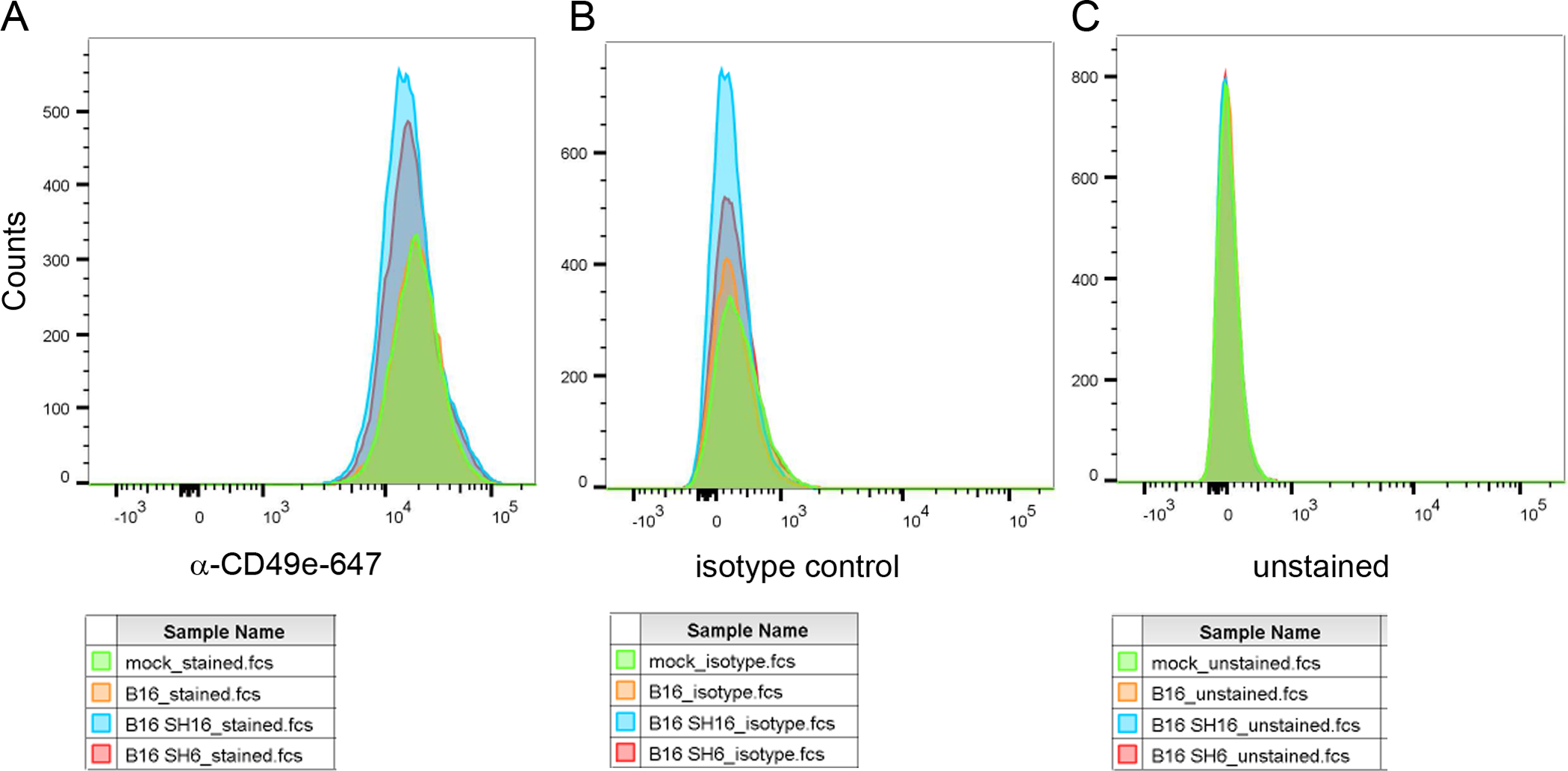

Supplement: S6 Fig — Histogram of cell surface α5 integrin expression in B16V, B16Vmock, B16VshUst(6) and B16VshUst(16) cell lines. Living cells were stained with (A) the antibody CD49e-Alexa647 or (B) the isotype control and subjected to FACS analysis. (C) Unstained cells were used as control. The histograms are one out of three representative experiments and display the same amount of α5 integrin on the cell surface of the 4 cell lines (n = 3). (TIF) [file pone.0170054.s006.tif]

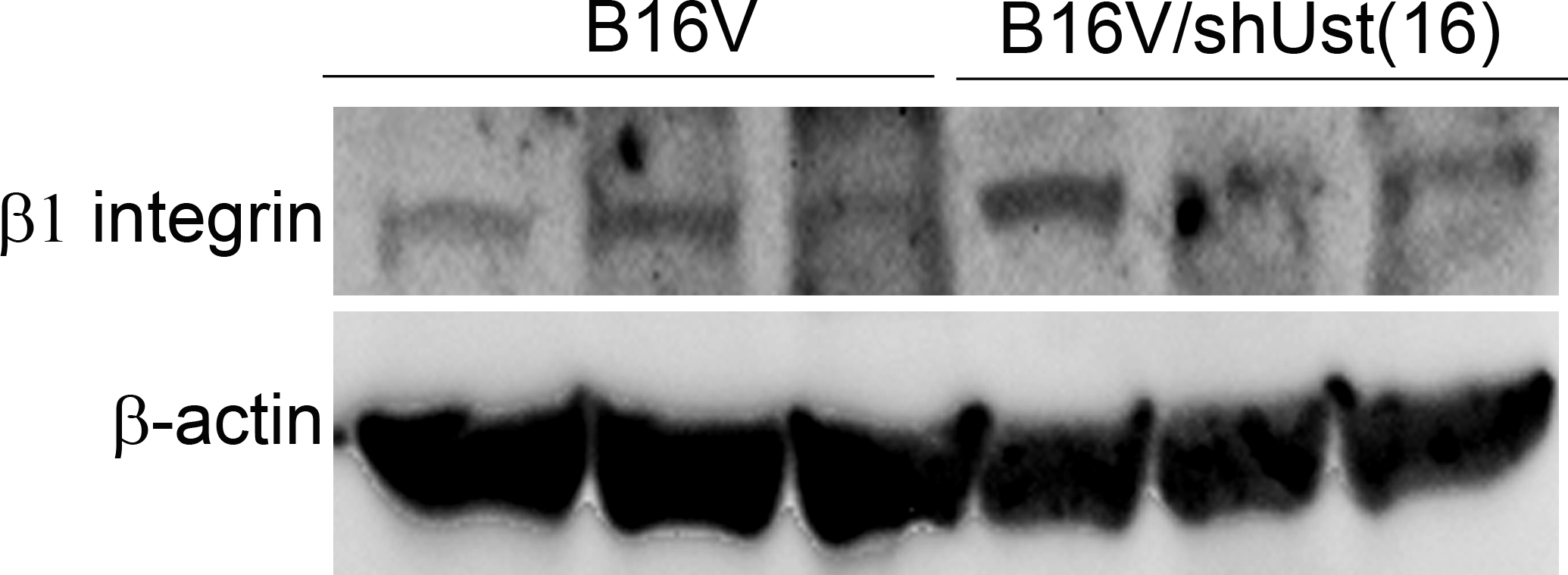

Supplement: S7 Fig — Immuno blots of three control and three B16VshUst(16) primary tumors lysates for β1 integrin and β-actin as loading control. The β1 integrin blot was used after stripping. Therefore, the loading control β-actin is the same as in Fig 5C. (TIF) [file pone.0170054.s007.tif]
